# Preoperative nutritional status and sarcopenia are associated with disease-free survival in patients with resected pancreatic neuroendocrine tumors

**DOI:** 10.3389/fonc.2026.1795586

**Published:** 2026-04-28

**Authors:** Jincheng Li, Mingyu Lai

**Affiliations:** Department of Gastroenterology, The First Affiliated Hospital of Guangxi Medical University, Nanning, Guangxi Zhuang, China

**Keywords:** body composition, computed tomography, disease-free survival, pancreatic neuroendocrine tumor, prognosis, prognostic nutritional index, sarcopenia, skeletal muscle index

## Abstract

**Background:**

Pancreatic neuroendocrine tumors (pNETs) show heterogeneous clinical behavior, and better predictors of postoperative recurrence are needed.

**Methods:**

We retrospectively analyzed 86 patients who underwent curative-intent resection for pNETs at a tertiary referral center. Preoperative nutritional status was assessed using the prognostic nutritional index (PNI), with a predefined literature-based cut-off of PNI < 50, and sarcopenia was defined on preoperative CT using L3 skeletal muscle index (SMI) and Prado sex-specific cut-offs. Disease-free survival (DFS) was evaluated using Kaplan-Meier methods and Cox regression.

**Results:**

Over a median follow-up of 21.8 months (IQR 7.7-37.2), 21 patients (24.4%) developed recurrence or progression. Sarcopenia was present in 47 patients (54.7%) and low PNI in 50 patients (58.1%), with 32 patients (37.2%) meeting both criteria. Sarcopenia was associated with shorter DFS (log-rank P = 0.034) and a higher hazard of recurrence in univariable analysis. After adjustment in a parsimonious multivariable model, this association was attenuated and no longer statistically significant.

**Conclusions:**

Preoperative CT-defined sarcopenia was associated with DFS in resected pNETs, but its independence from tumor burden remains uncertain and should be tested in larger prospective multicenter studies using standardized CT protocols.

## Introduction

Pancreatic neuroendocrine tumors account for a small proportion of pancreatic neoplasms but have shown an increasing incidence over recent decades (1, 2). Although surgical resection offers favorable long-term outcomes for many patients, postoperative recurrence remains clinically meaningful, and risk varies substantially between individuals. Tumor size and histological grade are established prognostic factors (3, 4), yet tumor-centric parameters alone do not fully explain outcome heterogeneity.

Increasing attention has therefore been directed toward host-related factors. The prognostic nutritional index (PNI), originally proposed by Onodera et al. (5), integrates serum albumin and lymphocyte count as a composite marker of nutritional and immune status. In pancreatic neuroendocrine neoplasms and related gastro-entero-pancreatic neuroendocrine tumor cohorts, lower PNI has been associated with poorer postoperative outcomes (6, 7).

Sarcopenia—loss of skeletal muscle mass—has also emerged as a prognostic marker in oncology (8, 9). CT-based assessment of skeletal muscle at the third lumbar vertebra (L3) is a validated and widely adopted approach for quantifying muscle mass (10). However, evidence focusing specifically on resected pNETs, particularly in Asian populations, remains limited.

Therefore, this study aimed to investigate the prognostic significance of preoperative PNI and CT-derived sarcopenia in a Chinese cohort of patients with surgically resected pNETs.

## Materials and methods

This retrospective single-center cohort study was conducted at The First Affiliated Hospital of Guangxi Medical University, a tertiary academic referral center. Consecutive patients with pathologically confirmed pancreatic neuroendocrine tumors (pNETs) who underwent curative-intent resection between June 2014 and May 2025 were screened. Management decisions were made in accordance with international guidelines and multidisciplinary assessment (11, 12). Tumors were staged according to the AJCC 8th edition TNM system and graded according to the WHO 2019 classification of digestive system tumors (13). The study was conducted in accordance with the Declaration of Helsinki and approved by the institutional ethics committee.

Preoperative work-up generally included contrast-enhanced cross-sectional imaging (CT and/or MRI) for staging and surgical planning. Preoperative laboratory tests were collected as part of routine care. The prognostic nutritional index (PNI) was calculated using the Onodera formula and categorized using a literature-based threshold (PNI < 50).

Skeletal muscle at the L3 level was segmented using 3D Slicer with a predefined attenuation threshold of -29 to 150 HU. Small islands were removed and morphological closing was applied to obtain a continuous region of interest. Non-muscle tissues, including air and bone, were manually excluded. Muscle volume and radiodensity parameters were extracted using the Segment Statistics module. The cross-sectional muscle area (SMA) was calculated by dividing the segmented volume by slice thickness, and the skeletal muscle index (SMI) was derived by normalizing SMA to height squared (cm²/m²). Mean muscle attenuation (HU) was used to assess muscle quality. Sarcopenia was defined using the Prado sex-specific cut-offs (male < 52.4 cm²/m²; female < 38.5 cm²/m²).

Patients were followed through scheduled radiologic evaluations and structured telephone interviews. DFS was defined as the interval from surgery to the first radiologically documented recurrence or progression. Patients without DFS events were censored at the date of the last available radiologic assessment; when imaging was performed outside our institution, the date of the most recent reported assessment was ascertained by structured telephone contact and used for censoring. Death without documented recurrence was censored at the last radiologic assessment.

Continuous variables are reported as median (interquartile range, IQR) and categorical variables as number (percentage). Group comparisons used the Mann-Whitney U test for continuous variables and Fisher exact test or chi-square test for categorical variables as appropriate. DFS was estimated using Kaplan-Meier methods with log-rank tests. Cox proportional hazards models were used for univariable and multivariable analyses. Given the limited number of DFS events, we prespecified a parsimonious multivariable model including age, sex, tumor size, stage (III-IV vs I-II), PNI category, and sarcopenia. Histological grade was not included in the primary multivariable model because no DFS events occurred among grade 1 tumors, leading to unstable estimation. Proportional hazards assumptions were assessed using Schoenfeld residuals, with no evidence of major violation. Three otherwise eligible cases with incomplete follow-up information were excluded before the final analytic dataset was locked; therefore, primary analyses were performed using complete-case data.

## Results

Baseline characteristics are summarized in **Table 1**. The cohort included 86 patients, with 21 DFS events (24.4%) over a median follow-up of 21.8 months (IQR 7.7-37.2). Stage distribution was I 19 (22.1%), II 22 (25.6%), III 26 (30.2%), and IV 19 (22.1%); stage III-IV accounted for 45 patients (52.3%). Histological grade was G1 in 40 patients (46.5%), G2 in 42 (48.8%), and G3/NEC in 4 (4.7%); notably, no recurrences were observed among grade 1 tumors (0/40). Functioning tumors comprised 19 patients (22.1%). Sarcopenia was present in 47 patients (54.7%), low PNI in 50 patients (58.1%), and 32 patients (37.2%) met both criteria.

**Table 1:** Baseline characteristics by DFS status.

Variable	No event (n=65)	Event (n=21)	P
Age (years)	49.00 (39.00–57.00)	52.00 (44.00–59.00)	0.260
BMI (kg/m²)	23.67 (22.04–26.30)	20.57 (19.38–22.27)	0.001
PNI	49.50 (46.15–52.35)	47.30 (45.65–50.90)	0.482
Tumor size (cm)	3.50 (2.00–4.90)	5.00 (3.80–7.00)	0.003
SMI (cm²/m²)	42.83 (35.39–51.03)	38.10 (29.43–44.27)	0.059
Sex			0.791
Female	44 (67.7%)	13 (61.9%)	
Male	21 (32.3%)	8 (38.1%)	
Low PNI (PNI < 50)			0.801
No	28 (43.1%)	8 (38.1%)	
Yes	37 (56.9%)	13 (61.9%)	
Sarcopenia (Prado)			0.026
No	34 (52.3%)	5 (23.8%)	
Yes	31 (47.7%)	16 (76.2%)	
Stage			<0.001
I	19 (29.2%)	0 (0.0%)	
II	19 (29.2%)	3 (14.3%)	
III	23 (35.4%)	3 (14.3%)	
IV	4 (6.2%)	15 (71.4%)	
Functioning pNET			0.139
No	48 (73.8%)	19 (90.5%)	
Yes	17 (26.2%)	2 (9.5%)	
Grade			<0.001
G1	40 (61.5%)	0 (0.0%)	
G2	24 (36.9%)	18 (85.7%)	
G3	1 (1.5%)	2 (9.5%)	
NEC	0 (0.0%)	1 (4.8%)	

**Figure 1:**
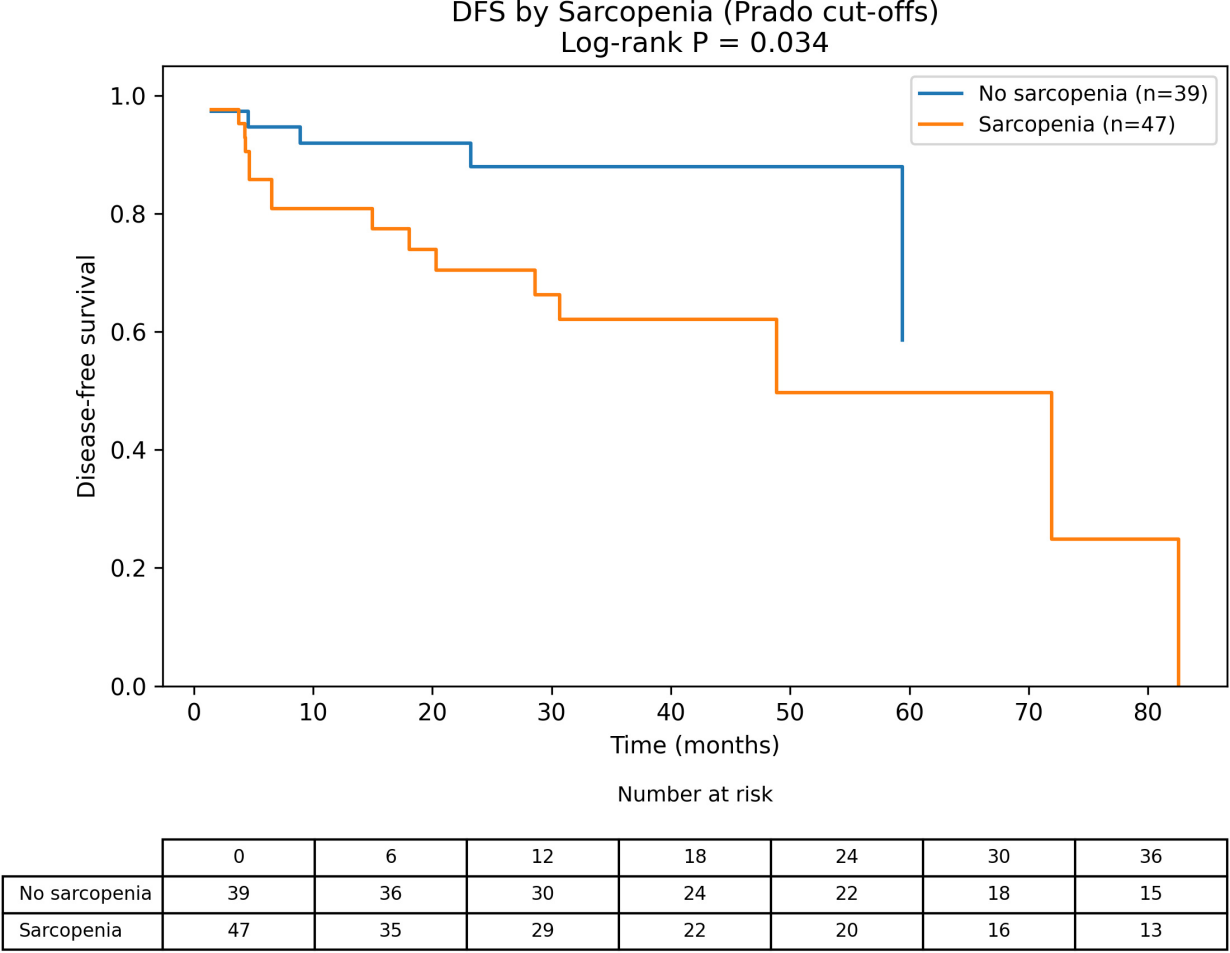
Disease-free survival stratified by CT-defined sarcopenia (Prado cut-offs). Kaplan–Meier curves of disease-free survival (DFS) comparing patients with and without sarcopenia. Skeletal muscle index (SMI) was measured on preoperative unenhanced CT at the third lumbar vertebra (L3) and normalized for height. Sarcopenia was defined using Prado sex-specific thresholds (male < 52.4 cm²/m²; female < 38.5 cm²/m²). Tick marks indicate censored observations. Differences between groups were assessed using the log-rank test (P = 0.034).

**Figure 2:**
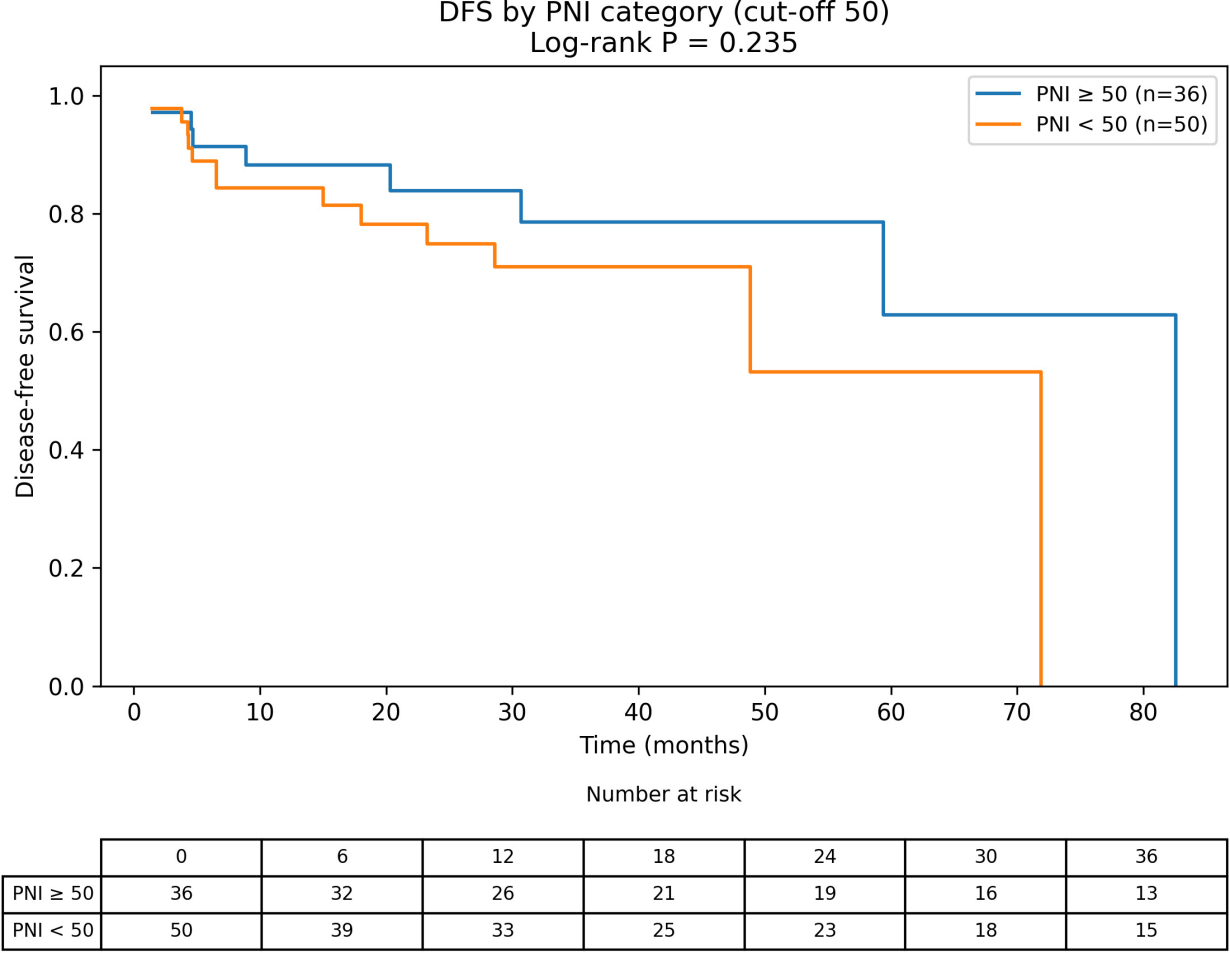
Disease-free survival stratified by prognostic nutritional index category (PNI < 50 vs. ≥ 50). Kaplan–Meier curves of DFS according to preoperative PNI category. PNI was calculated as 10×serum albumin (g/dL) + 0.005×total lymphocyte count (/mm³) and categorized using a literature-based threshold (PNI < 50). Tick marks indicate censored observations. Differences between groups were assessed using the log-rank test (P = 0.235).

Kaplan-Meier analysis showed significantly shorter DFS among patients with sarcopenia (log-rank P = 0.034; **Figure 1**). In contrast, DFS did not differ between low and high PNI groups using the PNI < 50 threshold (log-rank P = 0.235; **Figure 2**). In an additional four-group stratification by BMI (25 kg/m²) and sarcopenia status, the overall separation was not statistically significant (log-rank P = 0.137; **Supplementary Figure S1**). Among patients with DFS events, the median time to recurrence/progression was 8.9 months (IQR 4.5-28.6).

In univariable Cox regression (**Table 2**), sarcopenia was associated with DFS (HR 2.87, 95% CI 1.03-7.99, P = 0.043). Tumor size (HR 1.26, 95% CI 1.07-1.48, P = 0.006), stage III-IV versus I-II (HR 5.76, 95% CI 1.67-19.87, P = 0.006), BMI (HR 0.80, 95% CI 0.69-0.93, P = 0.004), and continuous SMI (HR 0.95, 95% CI 0.90-1.00, P = 0.040) were also associated with DFS. Low PNI (PNI < 50) was not associated with DFS (HR 1.75, 95% CI 0.69-4.48, P = 0.241). In the compact multivariable model (**Table 3**), stage III-IV showed a borderline association with DFS (HR 3.65, 95% CI 0.87-15.32, P = 0.077), while sarcopenia and low PNI were not independently significant.

**Table 2:** Univariable Cox regression for DFS.

Predictor	HR (95% CI)	P
Age (years)	1.026 (0.988–1.065)	0.181
Male sex	1.093 (0.446–2.682)	0.846
BMI (per 1 kg/m²)	0.802 (0.690–0.933)	0.004
Tumor size (per 1 cm)	1.257 (1.067–1.480)	0.006
Stage III–IV vs I–II	5.762 (1.671–19.874)	0.006
PNI (continuous)	0.951 (0.875–1.033)	0.232
Low PNI (PNI < 50)	1.753 (0.685–4.484)	0.241
SMI (continuous)	0.950 (0.904–0.998)	0.04
Sarcopenia (Prado)	2.873 (1.034–7.985)	0.043

**Table 3:** Multivariable Cox regression for DFS.

Predictor	HR (95% CI)	P
Age (years)	1.019 (0.977–1.062)	0.383
Male sex	1.055 (0.407–2.736)	0.912
Tumor size (cm)	1.105 (0.899–1.358)	0.344
Stage III–IV vs I–II	3.649 (0.869–15.323)	0.077
Low PNI (PNI < 50)	1.535 (0.571–4.122)	0.395
Sarcopenia (Prado)	2.163 (0.751–6.232)	0.153

## Discussion

In this retrospective cohort of patients with surgically resected pancreatic neuroendocrine tumors, tumor size and stage were strongly associated with disease-free survival, consistent with the established influence of tumor burden on postoperative recurrence risk. Although histological grade is also a recognized prognostic factor, its effect could not be modeled reliably in the primary multivariable analysis because no DFS events occurred among grade 1 tumors, leading to complete separation and unstable estimation.

Beyond tumor-related factors, CT-defined sarcopenia was associated with worse DFS in univariable analysis. Skeletal muscle is closely linked to metabolic homeostasis and immune competence, so reduced muscle mass may reflect limited physiologic reserve as well as tumor-related catabolism. After adjustment for tumor burden, however, the association weakened, suggesting that sarcopenia in this setting is more likely to represent an indicator of host frailty and disease burden than a fully independent determinant of recurrence risk.

When PNI was analyzed using the predefined literature-based threshold of PNI < 50, low PNI was not associated with DFS in our cohort. We now make a clearer distinction between this primary, literature-based analysis and any exploratory data-driven threshold analyses, which should be interpreted as hypothesis-generating only. Recent work in pancreatic ductal adenocarcinoma has proposed an albumin-myosteatosis gauge as a more integrated prognostic measure; whether a similar combined approach could improve risk stratification in pNETs deserves further study (14).

This study has several limitations. First, its retrospective single-center design may limit generalizability. Second, the median follow-up of 21.8 months is relatively short for a tumor type such as pNET, and late recurrences may therefore be underrepresented. Third, only 21 DFS events were observed, which limits model stability and leaves the multivariable estimates vulnerable to overfitting despite the use of a parsimonious model. Fourth, histological grade—one of the most important prognostic variables in pNET—could not be included in the primary multivariable model because complete separation occurred, and this should be regarded as a major limitation. Finally, although CT-based muscle quantity and attenuation were measured with a standardized workflow, body composition does not fully capture functional performance or broader frailty. Even so, the present findings support further evaluation of preoperative CT-derived sarcopenia as a clinically accessible prognostic marker in resected pNETs (15, 16).

## Data Availability

The data supporting the findings of this study are not publicly available because they contain information that could compromise the privacy of research participants. Summary data are included in the article and **Supplementary Material**. Requests to access de-identified data may be directed to the corresponding author, Mingyu Lai (2411423770@qq.com), and will be considered upon reasonable request and subject to institutional approvals.

## Supplementary material

Supplementary Figure S1 DFS by combined BMI (25 kg/m²) and sarcopenia status. Kaplan–Meier curves of DFS across four subgroups defined by BMI (< 25 vs. ≥ 25 kg/m²) and CT-defined sarcopenia (Prado cut-offs). Tick marks indicate censored observations. Overall group differences were assessed using the log-rank test (P = 0.137).
