# Supplementary figures and images for: Preoperative nutritional status and sarcopenia are associated with disease-free survival in patients with resected pancreatic neuroendocrine tumors
Source: Front Oncol. 2026 Apr 28;16:1795586. doi: 10.3389/fonc.2026.1795586 (PMC13160750; doi:10.3389/fonc.2026.1795586)

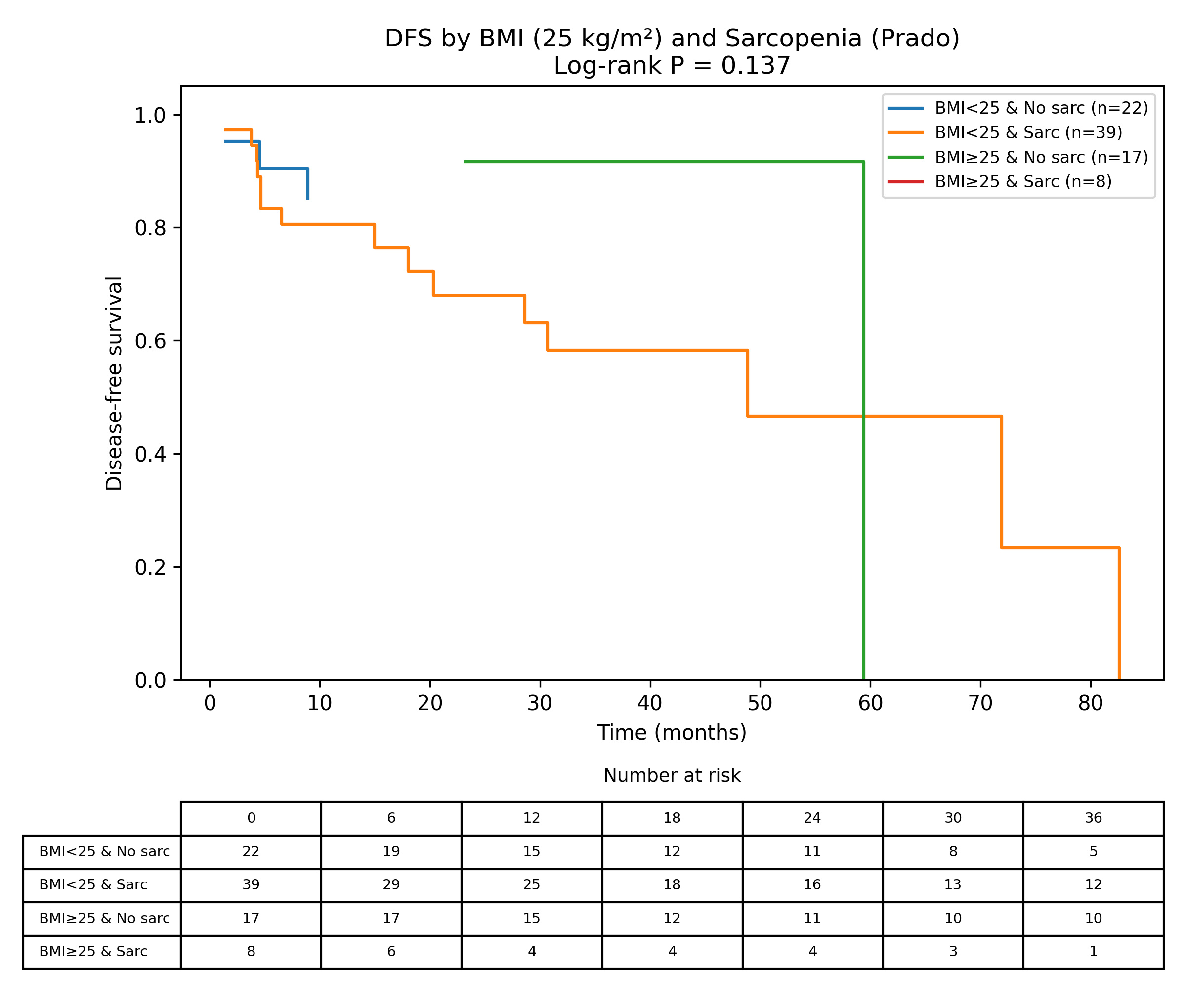

Supplement: Supplementary Figure S1 — DFS by combined BMI (25 kg/m²) and sarcopenia status. Kaplan–Meier curves of DFS across four subgroups defined by BMI (< 25 vs. ≥ 25 kg/m²) and CT-defined sarcopenia (Prado cut-offs). Tick marks indicate censored observations. Overall group differences were assessed using the log-rank test (P = 0.137). [file Image1.jpeg]
